# Supplementary material for: Nonalcoholic Fatty Liver Disease (NAFLD) and Hepatic Cytochrome P450 (CYP) Enzymes
Source: Pharmaceuticals (Basel). 2020 Aug 29;13(9):222. doi: 10.3390/ph13090222 (PMC7560175; doi:10.3390/ph13090222)
Supplement: Supplementary file 1 [file pharmaceuticals-13-00222-s001.pdf]

Nonalcoholic Fatty Liver Disease (NAFLD) and Hepatic Cytochrome P450 (CYP) Enzymes

Rohitash Jamwal * and Benjamin J. Barlock

Biomedical and Pharmaceutical Sciences, University of Rhode Island, Kingston, RI 02881, USA; bbarlock@my.uri.edu
* Correspondence: rohitash@my.uri.edu

Supplementary Table S1. Therapeutic agents currently being developed for the treatment of NAFLD. ACC, Acetyl-CoA Carboxylase; AOC, amine oxidase, copper containing; ASK, Apoptosis signal-regulating kinase; FGF, fibroblast growth factor; FXR, Farnesoid X receptor; PPAR, peroxisome proliferator-activated receptors; mTOT, mitochondrial target of thiazolidinediones; SGLT, Sodium-dependent glucose cotransporter; GLP, Glucagon-like peptide; ASBT, apical sodium–bile acid transporter; ASK, apoptosis signal-regulating kinase; THB, thyroid hormone receptor.

Therapeutic candidate	Company	Mechanism of action	Target	Ref.
GFT505	Genefit Pharmaceuticals	PPARα and δ agonist	Lipid metabolism	[1]
Obeticholic acid	Intercept Pharmaceuticals	FXR agonist	Lipid metabolism	[2]
BMS-986036	Bristol-Myers Squibb	FGF21 analog	Lipid metabolism	[3]
GS-0976	Gilead Sciences Inc.	ACC inhibitor	Lipid metabolism	[4]
PF-05221304	Pfizer Inc.	ACC inhibitor	Lipid metabolism	[5]
Selonsertib	Gilead Sciences Inc.	ASK1 inhibitor	Lipid metabolism	[6]
GR-MD-02	Galectin Therapeutics	Galectin inhibitor	Fibrosis	[7]
Aramchol	Galmed Pharmaceuticals	SCD1 inhibitor	Lipid metabolism	[8]
Cenicriviroc	Allergan plc	CCR2/5 antagonist	Inflammation/cell death	[9]
NGM282	NGM Biopharmaceuticals Inc.	FGF19 analogue	Lipid metabolism	[10]
Volixibat (SHP626)	Shire	ASBT inhibitor	Bile acid synthesis	[11]
LIK066	Novartis Pharmaceuticals	SGLT1 and 2 inhibitor	Glucose reabsorption	[12]
LJN452	Novartis Pharmaceuticals	FXR agonist	Lipid metabolism	[13]
MSDC 0602k	Cirius Therapeutics	mTOT modulator	Insulin signaling	[14]
Liraglutide (NN2211)	Novo Nordisk	GLP-1 analogue	Insulin signaling	[15]
Emricasan	Conatus and Novartis Pharmaceuticals	Pan-caspase inhibitor	Apoptosis	[16]
MGL-3196	Madrigal Pharmaceuticals	THR β-selective agonist	Lipid metabolism	[17]

1. Ratziu, V.; Harrison, S.A.; Francque, S.; Bedossa, P.; Lehert, P.; Serfaty, L.; Romero-Gomez, M.; Boursier, J.; Abdelmalek, M.; Caldwell, S., et al. Elafibranor, an Agonist of the Peroxisome Proliferator-

Activated Receptor-alpha and -delta, Induces Resolution of Nonalcoholic Steatohepatitis Without Fibrosis Worsening. *Gastroenterology* **2016**, *150*, 1147-1159 e1145, doi:10.1053/j.gastro.2016.01.038.

2. Neuschwander-Tetri, B.A.; Loomba, R.; Sanyal, A.J.; Lavine, J.E.; Van Natta, M.L.; Abdelmalek, M.F.; Chalasani, N.; Dasarathy, S.; Diehl, A.M.; Hameed, B., et al. Farnesoid X nuclear receptor ligand obeticholic acid for non-cirrhotic, non-alcoholic steatohepatitis (FLINT): a multicentre, randomised, placebo-controlled trial. *Lancet* **2015**, *385*, 956-965, doi:10.1016/S0140-6736(14)61933-4.
3. Sanyal, A.; Charles, E.D.; Neuschwander-Tetri, B.; Loomba, R.; Harrison, S.; Abdelmalek, M.; Lawitz, E.; Halegoua-DeMarzio, D.; Dong, Y.; Noviello, S., et al. BMS-986036 (pegylated FGF21) in patients with non-alcoholic steatohepatitis: a phase 2 study. *Journal of Hepatology* **2017**, *66*, S89-S90, doi:10.1016/S0168-8278(17)30443-9.
4. Lawitz, E.J.; Coste, A.; Poordad, F.; Alkhouri, N.; Loo, N.; McColgan, B.J.; Tarrant, J.M.; Nguyen, T.; Han, L.; Chung, C., et al. Acetyl-CoA Carboxylase Inhibitor GS-0976 for 12 Weeks Reduces Hepatic De Novo Lipogenesis and Steatosis in Patients With Nonalcoholic Steatohepatitis. *Clin Gastroenterol Hepatol* **2018**, 10.1016/j.cgh.2018.04.042, doi:10.1016/j.cgh.2018.04.042.
5. Bergman, A.; Gonzalez, S.C.; Tarabar, S.; Saxena, A.; Esler, W.; Amin, N. Safety, tolerability, pharmacokinetics and pharmacodynamics of a liver-targeting ACC inhibitor (PF-05221304) following single and multiple oral doses. *Journal of Hepatology* **2018**, *68*, S582, doi:10.1016/S0168-8278(18)31423-5.
6. Loomba, R.; Lawitz, E.; Mantry, P.S.; Jayakumar, S.; Caldwell, S.H.; Arnold, H.; Diehl, A.M.; Djedjos, C.S.; Han, L.; Myers, R.P., et al. The ASK1 inhibitor selonsertib in patients with nonalcoholic steatohepatitis: A randomized, phase 2 trial. *Hepatology* **2017**, 10.1002/hep.29514, doi:10.1002/hep.29514.
7. Harrison, S.A.; Marri, S.R.; Chalasani, N.; Kohli, R.; Aronstein, W.; Thompson, G.A.; Irish, W.; Miles, M.V.; Xanthakos, S.A.; Lawitz, E., et al. Randomised clinical study: GR-MD-02, a galectin-3 inhibitor, vs. placebo in patients having non-alcoholic steatohepatitis with advanced fibrosis. *Aliment Pharmacol Ther* **2016**, *44*, 1183-1198, doi:10.1111/apt.13816.
8. Hashmonai, M.; Schramek, A.; Better, O. Causes of early obstruction of sub-cutaneous A-V fistulae for haemodialysis. *Vasc Surg* **1974**, *8*, 36-40.
9. Friedman, S.L.; Ratziu, V.; Harrison, S.A.; Abdelmalek, M.F.; Aithal, G.P.; Caballeria, J.; Francque, S.; Farrell, G.; Kowdley, K.V.; Craxi, A., et al. A randomized, placebo-controlled trial of cenicriviroc for treatment of nonalcoholic steatohepatitis with fibrosis. *Hepatology* **2018**, *67*, 1754-1767, doi:10.1002/hep.29477.
10. Harrison, S.A.; Rinella, M.E.; Abdelmalek, M.F.; Trotter, J.F.; Paredes, A.H.; Arnold, H.L.; Kugelmas, M.; Bashir, M.R.; Jaros, M.J.; Ling, L., et al. NGM282 for treatment of non-alcoholic steatohepatitis: a multicentre, randomised, double-blind, placebo-controlled, phase 2 trial. *Lancet* **2018**, *391*, 1174-1185, doi:10.1016/S0140-6736(18)30474-4.
11. Tiessen, R.G.; Kennedy, C.A.; Keller, B.T.; Levin, N.; Acevedo, L.; Gedulin, B.; van Vliet, A.A.; Dorenbaum, A.; Palmer, M. Safety, tolerability and pharmacodynamics of apical sodium-dependent bile acid transporter inhibition with volixibat in healthy adults and patients with type 2 diabetes mellitus: a randomised placebo-controlled trial. *BMC Gastroenterol* **2018**, *18*, 3, doi:10.1186/s12876-017-0736-0.
12. HE, Y.; HAYNES, W.G.; MEYERS, C.D.; AMER, A.; ZHANG, Y.; MAHLING, P.C.; MENDONZA, A.E.; MA, S.; CHUTKOW, W.; BACHMAN, E.S. LIK066, a Dual SGLT1/2 Inhibitor, Reduces Weight and Improves Multiple Incretin Hormones in Clinical Proof-of-Concept Studies in Obese Patients With or Without Diabetes. *Diabetes* **2018**, *67*, doi:10.2337/db18-114-LB.
13. Tully, D.C.; Rucker, P.V.; Chianelli, D.; Williams, J.; Vidal, A.; Alper, P.B.; Mutnick, D.; Bursulaya, B.; Schmeits, J.; Wu, X., et al. Discovery of Tropifexor (LJN452), a Highly Potent Non-bile Acid FXR Agonist for the Treatment of Cholestatic Liver Diseases and Nonalcoholic Steatohepatitis (NASH). *J Med Chem* **2017**, *60*, 9960-9973, doi:10.1021/acs.jmedchem.7b00907.

14. Colca, J.R.; McDonald, W.G.; Adams, W.J. MSDC-0602K, a metabolic modulator directed at the core pathology of non-alcoholic steatohepatitis. *Expert Opin Investig Drugs* **2018**, *27*, 631-636, doi:10.1080/13543784.2018.1494153.
15. Armstrong, M.J.; Gaunt, P.; Aithal, G.P.; Barton, D.; Hull, D.; Parker, R.; Hazlehurst, J.M.; Guo, K.; team, L.t.; Abouda, G., et al. Liraglutide safety and efficacy in patients with non-alcoholic steatohepatitis (LEAN): a multicentre, double-blind, randomised, placebo-controlled phase 2 study. *Lancet* **2016**, *387*, 679-690, doi:10.1016/S0140-6736(15)00803-X.
16. Shiffman, M.; Freilich, B.; Vuppalanchi, R.; Watt, K.; Burgess, G.; Morris, M.; Sheedy, B.; Schiff, E. LP37 : A placebo-controlled, multicenter, double-blind, randomised trial of emricasan in subjects with non-alcoholic fatty liver disease (NAFLD) and raised transaminases. *Journal of Hepatology* **2015**, *62*, S282, doi:10.1016/S0168-8278(15)30191-4.
17. Harrison, S.; Moussa, S.; Bashir, M.; Alkhouri, N.; Frias, J.; Baum, S.; Tetri, B.; Bansal, M.; Taub, R. MGL-3196, a selective thyroid hormone receptor-beta agonist significantly decreases hepatic fat in NASH patients at 12 weeks, the primary endpoint in a 36 week serial liver biopsy study. *Journal of Hepatology* **2018**, *68*, S38, doi:10.1016/S0168-8278(18)30292-7.
